# Retraction Note: Exosome-transmitted miR-567 reverses trastuzumab resistance by inhibiting ATG5 in breast cancer

**DOI:** 10.1038/s41419-023-06254-5

**Published:** 2023-11-10

**Authors:** Mingli Han, Jianguo Hu, Pengwei Lu, Hui Cao, Chao Yu, Xiangke Li, Xueke Qian, Xue Yang, Yunqing Yang, Na Han, Dongwei Dou, Fan Zhang, Mulin Ye, Changcheng Yang, Yuanting Gu, Huaying Dong

**Affiliations:** 1 Department of Breast Surgery, The First Affiliated Hospital of Zhengzhou University, Zhengzhou, 450052 China; 2 Department of Obstetrics and Gynecology, The Second Affiliated Hospital, Chongqing Medical University, Chongqing, 400010 China; 3 Department of Vascular Surgery, The First Affiliated Hospital of Zhengzhou University, Zhengzhou, 450052 China; 4 Department of General Surgery, University-Town Hospital of Chongqing Medical University, Chongqing, 400016 China; 5 Department of Oncology, The First Affiliated Hospital of Zhengzhou University, Zhengzhou, 450052 China; 6 Department of General Surgery, Hainan General Hospital, Hainan Affiliated Hospital of Hainan Medical University, Haikou, 570311 China; 7 Department of Oncology, The First Affiliated Hospital of Hainan Medical University, Haikou, 570102 China

Retraction to: *Cell Death & Disease* 10.1038/s41419-020-2250-5, published online 22 January 2020

The Editors-in-Chief have retracted this article. After publication, concerns were raised regarding image similarities between this article and previous papers from different author groups, specifically:

- Two images in Fig. 4h appear highly similar to those in Fig. 1f of [1].
- The images in Fig. 7b appear highly similar to those in Fig. 8a of [2] with different cropping and rotation.
- The images in Fig. 8a appear highly similar to those in Figs. 4j and 5h of [3].

Further checks by the Publisher identified a number of further concerns:

- Fig. 4d SKBR-3-TR CQ+ GAPDH appears highly similar to Fig. 5d SKBR-3-TR Control GAPDH.
- Fig. 4d BT474-TR CQ- and CQ+ GAPDH appear highly similar to Fig. 5d BT474-TR Control and miR-567 GAPDH, respectively.
- Some of the blots in Figs. 7d and 8c appear to have irregularities in the backgrounds.
- The GSE104076 microarray dataset information [4] does not state that either cell group was resistant to trastuzumab, which makes it unsuitable for analysis of differential miRNAs between trastuzumab-resistant cells and -sensitive cells.

The Editors-in-Chief therefore no longer have confidence in the presented data and the conclusions of the article.

None of the authors have responded to any correspondence from the editor or publisher about this retraction.
